# Humidifier Disinfectant Consumption and Humidifier Disinfectant-Associated Lung Injury in South Korea: A Nationwide Population-Based Study

**DOI:** 10.3390/ijerph18116136

**Published:** 2021-06-06

**Authors:** Jeonggyo Yoon, Minsun Kang, Jaehun Jung, Min Jae Ju, Sung Hwan Jeong, Wonho Yang, Yoon-Hyeong Choi

**Affiliations:** 1 Department of Preventive Medicine, Gachon University College of Medicine, Incheon 21999, Korea; wjdry1234@gmail.com (J.Y.); eastside1st@gmail.com (J.J.); cconglove22@gmail.com (M.J.J.); 2 Artificial Intelligence and Big-Data Convergence Center, Gachon University Gil Medical Center, Incheon 21565, Korea; ms_kang127@naver.com; 3 Department of Health Sciences and Technology, GAIHST, Gachon University, Incheon 21999, Korea; 4 Division of Pulmonary and Critical Care Medicine, Department of Internal Medicine, Gachon University Gil Medical Center, Incheon 21565, Korea; jsw@gilhospital.com; 5 Department of Occupational Health, Daegu Catholic University, Gyeongbuk 38430, Korea; whyang@cu.ac.kr

**Keywords:** humidifier disinfectant, humidifier disinfectant sales, NHIS, HDLI, lung injury

## Abstract

Humidifier disinfectant (HD) is a household biocidal product used in humidifier water tanks to prevent the growth of microorganisms. In 2011, a series of lung injury cases of unknown causes emerged in children and pregnant women who had used HD in Korea. This study investigated changes in the nationwide number of cases of humidifier disinfectant-associated lung injury (HDLI) in concordance with nationwide HD consumption using data covering the entire Korean population. More than 25 kinds of HD products were sold between 1994 and 2011. The number of diagnosed HDLI, assessed by S27.3 (other injuries of lungs) of the Korea National Health Insurance Service (NHIS) data, sharply increased by 2005, subsequently decreased after 2005, and almost disappeared after 2011 in concordance with the annual number of HD sales. The number of self-reported HDLIs, assessed using data from all suspected HDLI cases registered in the Korea Ministry of Environment, changed with the annual number of HD sales, with a delay pattern, potentially induced by the late awareness of lung injury diseases. The present study suggests that changes in the nationwide annual consumption of HD products were consistent with changes in the annual number of HDLI cases in Korea.

## 1. Introduction

In the spring of 2011, a series of lung injury cases of unknown cause emerged in South Korea, identified by their association with the use of household chemicals, namely humidifier disinfectant (HD) [1,2]. To date, a total of 4114 people with HD-associated diseases (e.g., lung disease, asthma, and pneumonia), including 997 deaths, have been reported [3], which is known to be one of the worst environmental catastrophes to ever have occurred in Korea. HD is a biocidal product used in humidifier water tanks to prevent the growth of microorganisms, harmful bacteria, mold, and white dust. The major chemical ingredients used as HDs are polyhexamethylene guanidine (PHMG), chloromethylisothiazolinone/methylisothiazolinone (CMIT/MIT), and oligo-(2-(2-ethoxy)-ethoxyethyl) guanidine chloride (PGH), although major chemicals may differ depending on the product [4,5,6]. After the first HD product was commercially launched on the market in 1994, approximately 9.5 million products with 25 or more kinds of HD products were estimated to have been sold until the government announced the recall of all HD products in November 2011 [7]. Given such an amount of HD sales, a large number of people might be exposed to HD, and the adverse health effects might be more serious than reported. A recent study conducted by Byeon et al. (2016) estimated that 8.94 million people were exposed to HDs, and 0.95 million people experienced adverse health effects [8].

A growing body of evidence from in vitro [9,10] and in vivo [11,12] studies demonstrated that exposure to PHMG, CMIT/MIT, and PGH chemicals causes apoptosis, inflammation, and reactive oxygen stress in alveolar epithelial cells and induces airway barrier injuries, thereby leading to pulmonary inflammation and lung fibrosis. In addition, matched case-control studies (i.e., hospital-based, family-based, and community-based case-control studies) reported consistent findings of significant associations between the levels of HD exposure and the risk of a fatal lung injury called HD-associated lung injury (HDLI) [13,14,15]. Despite the potential role of PHMG, CMIT/MIT, and PGH in the pathogenesis of fatal lung injury as well as emerging evidence from case-control studies, the nationwide contribution of exposure to HD-related synthetic chemicals to the lung injury epidemic remains underappreciated. Unfortunately, it may not be appropriate to conduct epidemiological studies using a sample of the Korean general population due to the low incidence rate of disease in the general population as well as the particular characteristic that “most” of the exposed people developed disease. In the present study, to investigate the temporal changes in the nationwide HDLI cases in concordance with the nationwide HD sales, we evaluated the nationwide annual incidence of (1) diagnosed HDLI cases, which was recorded in the Korea National Health Insurance Service (NHIS) database, which covers the entire Korean population, and (2) self-reported HDLI cases using the exposure assessment database of all suspected cases registered in the Korea Ministry of Environment. We examined whether the annual incidences of HDLI cases have changed depending on the volume of HD sales, that is, total sales, sales by product, and sales by chemical type.

## 2. Materials and Methods

This study was conducted using three different data: (1) data of annual sales of humidifier disinfectant (HD), (2) data from the National Health Insurance Service (NHIS), and (3) data of the assessment of environmental exposure to HD. All methods were carried out in accordance with relevant guidelines and regulations. The second set of data was reviewed and approved by the Institutional Review Board of the Gachon University Gil Medical Center, which waived the need for consent (IRB No. GCIRB2019-232). The third set of data was reviewed and approved by the Institutional Review Board of Daegu Catholic University, and informed consent was obtained from all participants and a parent and/or legal guardians of children under 18 years old before participation (IRB No. CUIRB-2016-0114).

### 2.1. Annual Sales of HD

Data regarding annual sales from 1994 to 2011, i.e., launched year to banned year, were used for all available HD products. These data were obtained by combining data from the Asian Citizen’s Center for Environment and Health (ACCEH) [7], the Korea Centers for Disease Control and Prevention (KCDC) [16], the National Assembly, and Choi’s study [17]. Among the initial 25 kinds of HD products, those products with no available information on the sales period (*n* = 5) and annual sales (*n* = 3) were excluded from the analysis, leaving a total of 17 eligible HD products (see Supplementary Figure S1 online). Because HD products had been recalled since the Korean government officially banned the sales of HD in November 2011, we assigned a value by subtracting the annual recalls from the annual sales. When the number of annual recalls was larger than the number of annual sales, we assigned the value “0”.

Moreover, annual HD sales were classified by four chemical types: PHMG (CAS number: 89697-78-9), CMIT/MIT (CAS number: 26172-55-5/2682-20-4), PGH (CAS number: 374572-91-5), and other chemicals; (1) PHMG chemicals include the products of Oxy Ssakssak New Gaseupgi Dangbun (Oxy; Oxy Reckitt Benckiser (RB) Korea, Seoul, Korea), Lotte WiseLect Gaseupgi Salgyunje (Lotte; Lotte Mart, Seoul, Korea), Homeplus Gaseupgi Chungjungje (Homeplus; Homeplus, Seoul, Korea), and Homecare Bejiteobeulhom Gaseupgi Cleanup (Homecare; Costco Korea, Gyeonggi, Korea); (2) CMIT/MIT chemicals include the products of Aekyung Gaseupgi Mate and Palanhaneul Malg-eun Gaseupgi (Aekyung; Aekyung, Seoul, Korea), E-Mart Eplus Gaseupgi Salgyunje (E-Mart; E-Mart, Seoul, Korea), GS Hambakwussom Gaseupgi Sejeongje (GS; GS Retail, Seoul, Korea), Daiso Sandokkaebi Gaseupgi Punisher (Daiso; Asung Daiso, Seoul, Korea), Henkel HomeKeeper Humidifier Sterilizer (Henkel; Henkel Homecare Korea, Seoul, Korea), and SK Yugong Gaseupgi Mate; (3) PGH chemicals include the products of Butterfly Effect Cefu Gaseupgi Salgyunje (Butterfly Effect; Butterfly Effect, Gyeonggi, Korea) and Ato Organic Gaseupgi Salgyunje (Ato Organic; Ato Organic, Gyeonggi, Korea); (4) other chemicals include the products of Clannad N-with (Clannad; Clannad, Gyeonggi, Korea), Oxy Ssakssak with solid type (Oxy with solid type), Oxy Gaseupgi Dangbun, LG 119 Gaseupgi segyunjegeo, Ato Safe Gaseupgi Chungjungje/Hangkyunje (Ato Safe; Ato Safe, Seoul, Korea), Eco-fresh Gaseupgi Hangkyunball (Eco-fresh; JnK Science, Seoul, Korea), and Water&People Modern Life Salgyunball (Water&People; Nutricare, Gyeonggi, Korea) [16].

### 2.2. National Health Insurance Service (NHIS)-Recorded HDLI Cases

The diagnosed HDLI cases were identified from the NHIS database for the period of 1 January 2002 to 31 December 2017. The NHIS database is nationwide healthcare reimbursement data that covers the entire Korean population, including all citizens and long-term residents. The NHIS database includes personal information and healthcare utilization records of inpatient and outpatient usage (i.e., diagnoses, drug prescription, services received, and treatment cost) [18]. In the current study, because HDLI was a disease of unknown cause and a unique disease code was not available, we selected HDLI patients using potential diagnosis codes of J68.4, J84.9, J95.2, and S27.3 based on the International Statistical Classification of Diseases and Related Health Problems, 10th edition (ICD-10), where J68.4 indicates “Chronic respiratory conditions due to chemicals, gases, fumes and vapors”; J84.9 indicates “Interstitial pulmonary disease, unspecified”; J95.2 indicates “Acute pulmonary insufficiency following nonthoracic surgery”; and S27.3 indicates “Other injuries of lung.” A total of 176,025 newly diagnosed HDLI cases were identified in this study. Because the NHIS database was publicly available since 2002, it should be noted that NHIS-recorded HDLI cases in 2002 could not rule out pre-existing HDLI cases before 2002.

### 2.3. Self-Reported HDLI Cases from Exposure Assessment Database

Self-reported HDLI cases were identified by exposure assessment of HD. Exposure assessment of HD is a nationwide ongoing exposure assessment series designed to evaluate HD-related exposures and their health effects, which were conducted by the KCDC of the Korea Ministry of Health and Welfare between July 2013 and March 2014 and subsequently conducted by the Korea Environmental Industry and Technology Institute of Korea Ministry of Environment between July 2014 and the present [3]. Exposure assessment was performed for all registered participants who experienced clinical signs suggestive of HDLI that aimed to cover the entire exposed population in Korea and was carried out through extensive household interviews using structured HD-specific questionnaires regarding demographic characteristics, environmental exposure, and HD-related information [19]. The present study used the data of 6059 subjects enrolled in the four cycles of exposure assessment from July 2013 to May 2019 (360 subjects in cycle I, 168 subjects in cycle II, 669 subjects in cycle III, and 4862 subjects in cycle IV). In particular, cycle IV of the HD exposure assessment included four units, that is, IV-1, IV-2, IV-3, and IV-4. Because the last unit is still ongoing, we used all of the currently available data, and a total of 6059 subjects enrolled between cycles I and IV-3. The enrolled subjects who experienced clinical signs suggestive of HDLI were categorized as either current survivors or non-survivors (hereinafter referred to as HDLI-survivors or HDLI-deaths). HDLI-survivors were asked the question, “Which year was it when HDLI happened?”, while HDLI-deaths were asked, “Which year was it when the subjects died?”.

### 2.4. Statistical Analysis

All statistical analyses were performed using the SAS survey procedure (version 9.4; SAS Institute Inc., Cary, NC, USA). The statistical significance level was set at *p* < 0.05. We divided the NHIS-recorded HDLI cases into four diagnosis codes (J68.4, J84.9, J95.2, and S27.3) to evaluate the annual incidence of each diagnostic code. To calculate the incidence rate per 100,000 population for each year, the number of disease-specific cases for the year was divided by the number of populations for the year and multiplied by 100,000. Using the same method, the number of disease-specific cases stratified by age and sex for the year was divided by the number of populations stratified by age and sex for the year and multiplied by 100,000 to calculate the age- and sex-specific incidence rates per 100,000 population for each year. The total number of age- and sex-specific populations per year between 2002 and 2017 used the resident population data reported by Statistics Korea. The Spearman’s rank correlation coefficients were computed to evaluate the time-lag effects of annual HD sales (i.e., total sales, sales by product, and sales by chemicals) on the number of NHIS-recorded HDLI cases and self-reported HDLI cases using 0-to-5 year lag time. For self-reported HDLI cases, the Chi-square test was used to compare differences in the characteristics between HDLI-survivors and HDLI-deaths.

## 3. Results

Table 1 shows the annual sales of each HD product between 1994 and 2011. Regarding total sales of HD products, 9,482,014 products were sold. The annual sales of all HD products rapidly increased until 2005 (941,976 products in 2005) and gradually decreased in the following four years. During the entire sales period, the product of the highest HD sales was Oxy (Oxy New Gaseupgi Dangbun: 4,171,662 products (44.0%)), followed by Aekyung (Aekyung Gaseupgi Mate: 1,686,826 products (17.8%)).

### 3.1. Annual HD Sales and NHIS-Recorded HDLI Cases

Table 2 shows the annual incidence rate per 100,000 population of NHIS-recorded cases identified using the codes J68.4, J84.9, J95.2, and S27.3 for the period of 2002 to 2017. The absolute number of annual NHIS-recorded cases is shown in Supplementary Table S1 online. During this period, the annual incidence rates of J68.4 and J84.9 showed a gradual increase, the annual incidence rate of J95.2 showed irregular fluctuation, and the annual incidence rate of S27.3 showed an increase by 2005 and a decrease after 2005. In particular, the annual incidence rates of J84.9 and S27.3 differ depending on age and sex, respectively. The annual incidence rate of J84.9 dropped after 2011 in children (0–6 years), but steadily increased in other age groups (≥7 years). The annual incidence rate of S27.3 was higher in males than in females across all periods. 

**Figure 1:**
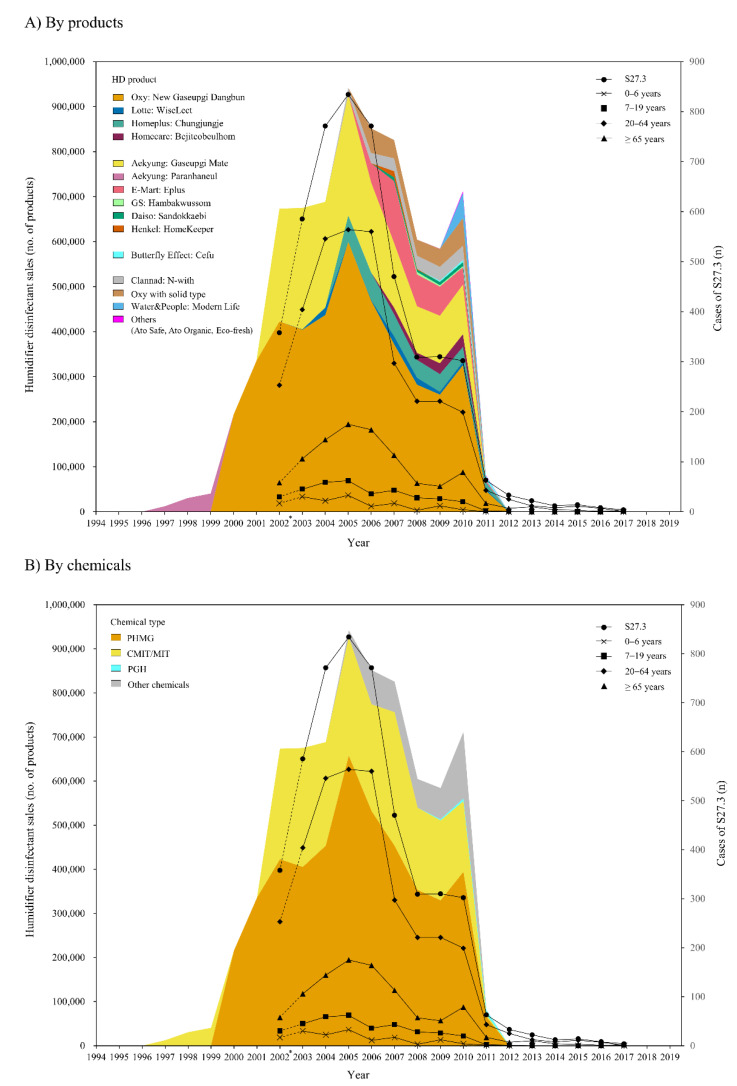
Annual sales of humidifier disinfectants and NHIS-recorded cases of S27.3. * Cases in 2002 may include pre-existing cases and are indicated as dotted lines.

Figure 1 shows the annual changes in HD sales and the number of S27.3 cases. The number of annual S27.3 cases and HD sales showed consistent patterns; total HD sales, HD sales of Oxy and Aekyung (by products), and HD sales of PHMG and CMIT/MIT (by chemicals) rapidly increased until 2005 and gradually decreased after 2005. Similarly, S27.3 cases increased until 2005 and decreased after 2005. In fact, significant positive correlations were observed between annual total HD sales and the annual number of NIHS-recorded S27.3 cases at a concurrent year to 2 years later, and the strongest such correlation was observed in the concurrent year (correlation coefficients, *r* = 0.907, 0.796, and 0.649 at lag of 0, 1, and 2 years, respectively, all *p*-values <0.01) (Table 3). Annual J68.4, J84.9, and J95.2 cases were not consistent with changes in annual HD sales (see Supplementary Figures S2–S4 online).

### 3.2. Annual HD Sales and Self-Reported HDLI Cases

Table 4 shows the general characteristics of all self-reported HDLI cases in Korea (*n* = 6059). Overall, 3102 subjects (51.2%) were males, and 2927 subjects were females (48.3%). Regarding age distribution, 8.5% of them were aged 0–6 years, 25.3% aged 7–19 years, 48.7% aged 20–64 years, and 17.5% aged ≥65 years. The most frequently used HD product in self-reported HDLI subjects was Oxy (75.9%), followed by Aekyung (8.5%) and Lotte (2.1%). HDLI-survivors were 4754 subjects (78.5%), while HDLI-deaths were 1305 subjects (21.5%). HDLI-survivors compared to HDLI-deaths were more likely to be females and highly educated, and the distributions of age and the most frequently used HD products significantly differed in HDLI-survivors and HDLI-deaths. Particularly, a proportion of children (0–6 years) and older adults (≥65 years) was higher in HDLI-deaths than HDLI-survivors. Additionally, the most frequently used HD product was reported as oxy in both HDLI-survivors and HDLI-deaths, while the second one was reported as Aekyung in HDLI-survivors and as unknown in HDLI-deaths.

**Figure 2:**
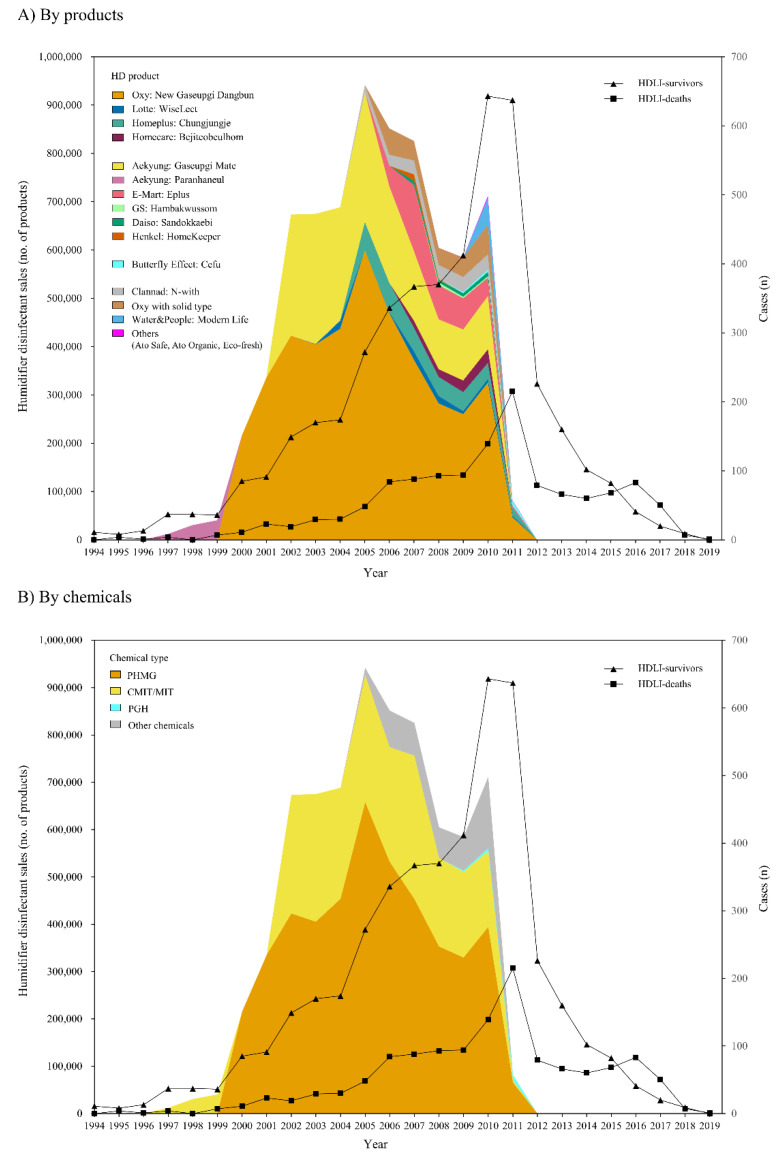
Annual sales of humidifier disinfectants and self-reported cases of HDLI.

Figure 2 shows the annual changes in HD sales and the number of self-reported HDLI cases. The number of annual HDLI-survivors and HDLI-deaths showed delayed patterns from the annual HD sales; total HD sales, HD sales of Oxy and Aekyung (by products), and HD sales of PHMG and CMIT/MIT (by chemicals) increased rapidly until 2005 and gradually decreased after 2005, while HDLI-survivors and HDLI-deaths increased until 2010 and 2011, respectively, and decreased in the following years. In fact, significant correlations were observed between annual total HD sales and the annual number of self-reported HDLI-survivor cases at the concurrent year to 5 years later (all *p*-values < 0.01), and the strongest such correlation was observed in HDLI-survivor cases 3 years later (*r* = 0.925). In addition, correlations were observed between annual total HD sales and annual number of self-reported HDLI-deaths at 1 to 5 years later (all *p*-values < 0.01), and the strongest such correlation was observed in HDLI-deaths 5 years later (*r* = 0.895) (Table 5).

## 4. Discussion

In the current study, we found that changes in nationwide annual consumption of HD products were consistent with changes in the number of HDLI cases, as observed in the assessment with diagnosed cases and self-reported cases using data from the Korea National Health Insurance Service (NHIS) and data from the Korea Ministry of Environment, which were able to reflect the whole Korean population. A total of annual HD sales had the strongest correlation with annual diagnosed HDLIs at the concurrent year (using data from the Korea NHIS, Figure 1 and Table 3) and with self-reported HDLIs 3 years later (using survivors’ data from the Korea Ministry of Environment, Figure 2 and Table 5). When we evaluated the chemical types of HD products, the consumption of PHMG-products, i.e., the most common type of HD chemicals, was consistent with the change in the number of HDLI cases, and changes in the consumption of CMIT/MIT-products, the second most common type of HD chemicals, was consistent with the changes in the number of HDLI cases. 

The most important pathological feature of HDLI cases is lung injury accompanied by inflammation and fibrosis, leading to various pulmonary diseases (e.g., asthma, pneumonia, and interstitial lung diseases) [20,21,22]. The major chemical components of HD products were reported as PHMG, CMIT/MIT, or PGH, although they may differ depending on the products, and recent experimental evidence has suggested that exposure to each of these chemicals is capable of inducing inflammation and leading to elevation in the fibrotic response. First, two in vivo studies using rats suggest that both inhalation and intratracheal instillation exposure to PHMG induce the release of pro-inflammatory cytokines, mRNA expression of fibronectin, and histopathological changes in lung tissues [12,23]. Another in vivo study in mice reported that intratracheal instillation of PGH also causes an increase in the levels of cytokines and fibronectin [24]. Second, two in vitro studies suggest that PHMG and CMIT/MIT induce the release of pro-inflammatory cytokines in mouse macrophage cells and mouse alveolar epithelial cells, respectively [9,25]. In addition, a study of human alveolar epithelial cells showed that exposure to PHMG and PGH elevates the level of the epithelial–mesenchymal transition (EMT)-related protein, an indicator of fibrotic response [10]. Additionally, there is a possibility for the role of microbiome advances in lung injuries and idiopathic pulmonary fibrosis (IPF), which may potentially link to the environmental exposures [26,27,28]. Taken together, it seems plausible that HD-induced inflammation and mRNA expression of fibronectin might trigger a wound-healing response, causing epithelial cells to be stiff and thickened, eventually leading to pulmonary fibrosis, which is identical to the features of HDLI cases. Although a few small-scale studies of matched case-control design support the above experimental findings and suggest significant associations with HD exposure and an increased risk of HDLI, there were still limited large-scale epidemiological studies [13,14,15].

The current study observed consistent patterns between annual HD sales and annual diagnosed HDLI in data from the NHIS (Figure 1). The number of HDLI cases, assessed by S27.3 cases (other injuries of lungs), was observed to sharply increase by 2005 and subsequently decrease after 2005 with an increase and decrease in the total number of HD sales, respectively (Figure 1). Their sales were banned in 2011, and thereafter, S27.3 cases almost disappeared. In fact, the number of S27.3 cases was 358 in 2002, 834 in 2005 (more than double), 63 in 2011 (7.55% of the highest year), and 4 in 2017 (0.48% of the highest year) (Table 2). However, annual total HD sales were not observed to be consistent with other diagnosed HDLIs, as assessed from the J68.4, J84.9, and J95.2 cases, which were assigned to patients who had chronic respiratory conditions due to chemicals, gases, fumes, and vapors; patients who had unspecified interstitial lung diseases; and patients who had acute or subacute development of cough, dyspnea, and breathlessness after nonthoracic surgery. Although HDLI may have clinical features of these three codes, our null observation may be explained by the possibility that HDLI patients were more likely to be assigned to S27.3 (other lung injuries) rather than the three codes, due to a structural problem of ICD-10 classification. HDLI was reported to be a series of lung injury cases of unknown cause before 2011 [1,2]. Thus, it might be difficult to use a conclusive diagnosis such as J68.4 until it is revealed that the HD exposure is a cause, and eventually S27.3, a kind of “garbage code”, might be assigned [29].

Interestingly, the current study observed delayed patterns between annual total HD sales and self-reported HDLI cases based on data registered in the Korea Ministry of Environment, which may reflect all suspected HDLI cases in Korea (Figure 2). The annual total HD sales had rapidly increased until 2005 and gradually decreased after 2005, while the annual number of self-reported HDLI incidences in survivors and HDLI-deaths increased by 2010 and 2011, respectively, and decreased in the following years (Figure 2). Our observed delayed pattern in self-reported HDLI incidence may be potentially caused by the late awareness of lung injury diseases, and a more delayed pattern in death cases might reflect the time span to reach death due to lung diseases. A few qualitative studies have reported that patients with lung diseases such as asthma [30], chronic obstructive pulmonary disease (COPD) [31], and IPF [32] had delayed self-awareness and were struggling to undergo diagnosis due to various reasons such as uncertainty of symptoms, minimization, or under-recognition of the severity of the symptoms, and misdiagnosis. 

The most important strength of this study is the use of two different data, which 1) may cover all potential HDLI cases in Korea and 2) may complement each other. The NHIS database includes national insurance claims data of the entire Korean population, capable of reflecting the nationwide incidence of lung injury and objective information using physician diagnosis, while lung injury cases recorded in the NHIS include HDLIs as well as other lung injuries. However, all suspected HDLI cases registered in the Korea Ministry of Environment could better explain lung injury cases, particularly those caused by HD exposure, while the HD-exposed population database may have inaccuracies due to the self-reported data. 

Nevertheless, the current study has several limitations. First, the nature of an ecological design study may not infer a causal relationship between HD sales and lung injury. Additionally, there were no data for the individual purchase of HDs, and we could not examine logistic regressions that may be a better model to examine associations between HDs and HDLIs. Second, the volume of HD “sales” in this study may not directly reflect the volume of HD “uses.” Third, the number of HD products with low sales volume was not included because of the lack of information on annual sales. Fourth, because HDLI does not have a unique disease code in the NHIS database and we used potential diagnoses with four ICD-10 codes, misclassification in HDLI diagnosis could not be ruled out. However, such an error is likely to be non-differential and leads to an estimated correlation toward the null. Thus, the actual correlation between annual HD sales and annual diagnosed HDLIs might be higher than our observation. Finally, we cannot rule out the possibility that annual changes in air pollution may affect the annual changes in the volume of lung injury, because previous studies reported that air pollution is associated with lung injuries such as IPF [33,34], interstitial lung disease [35], and pneumonia [36]. 

## 5. Conclusions

The current study suggests that changes in nationwide annual consumption of HD products were consistent with the changes in the annual number of HDLI cases in Korea. Given that HD exposure is epidemic in Korea and owing to controversial and limited evidence for the relationship between HD and lung injury, our findings contribute to epidemiological evidence that HD exposure is associated with the development of severe lung injury cases in Korea.

## Figures and Tables

**Table 1:** Annual sales of humidifier disinfectants during 1994–2011.

Chemical Type Product Name	Humidifier Disinfectant Sales, No. of Products																		
	Sum	1994	1995	1996	1997	1998	1999	2000	2001	2002	2003	2004	2005	2006	2007	2008	2009	2010	2011
All	9,482,014	0	0	0	12,325	30,661	40,714	215,865	335,376	673,579	675,071	688,500	941,976	851,808	825,779	604,738	584,391	712,076	81,568
PHMG	4,619,732	0	0	0	0	0	0	215,865	335,376	422,898	405,543	453,800	657,988	531,919	453,408	353,168	329,799	394,417	65,551
Oxy: New Gaseupgi Dangbun	4,171,662	0	0	0	0	0	0	215,865	335,376	422,898	404,530	437,222	599,637	469,167	372,357	282,172	260,144	325,533	46,761
Lotte: WiseLect	68,839	0	0	0	0	0	0	0	0	0	1013	16,578	0	1554	17,406	15,115	6207	7647	3319
Homeplus: Chungjungje	297,311	0	0	0	0	0	0	0	0	0	0	0	58,351	61,198	49,405	40,091	39,688	33,107	15,471
Homecare: Bejiteobeulhom	81,920	0	0	0	0	0	0	0	0	0	0	0	0	0	14,240	15,790	23,760	28,130	0 ^a^
CMIT/MIT	2,538,818	0	0	0	12,325	30,661	40,714	0	0	250,681	269,528	234,700	268,868	242,299	303,075	186,533	180,948	160,190	4695
Aekyung: Gaseupgi Mate	1,686,826	0	0	0	0	0	0	0	0	250,681	269,528	234,700	268,868	199,975	143,732	103,113	105,849	110,380	0
Aekyung: Palanhaneul	83,700	0	0	0	12,325	30,661	40,714	0 ^a^	0 ^a^	0 ^a^	0 ^a^	0 ^a^	0	0	0	0	0	0	0
E-Mart: Eplus	357,958	0	0	0	0	0	0	0	0	0	0	0	0	42,324	137,363	70,853	64,479	38,296	4643
GS: Hambakwussom	11,564	0	0	0	0	0	0	0	0	0	0	0	0	0	193	5581	3190	2548	52
Daiso: Sandokkaebi	29,576	0	0	0	0	0	0	0	0	0	0	0	0	0	8395	4785	7430	8966	0 ^a^
Henkel: HomeKeeper	15,593	0	0	0	0	0	0	0	0	0	0	0	0	0	13,392	2201	0 ^a^	0 ^a^	0 ^a^
SK: Yugong	353,601	NA ^b^	NA	NA	NA	NA	NA	NA	NA	0	0	0	0	0	0	0	0	0	0
PGH	19,812	0	0	0	0	0	0	0	0	0	0	0	0	0	0	0	2771	5996	11,045
Butterfly Effect: Cefu	17,414	0	0	0	0	0	0	0	0	0	0	0	0	0	0	0	2708	4625	10,081
Ato Organic	2398	0	0	0	0	0	0	0	0	0	0	0	0	0	0	0	63	1371	964
Other chemicals	2,303,652	0	0	0	0	0	0	0	0	0	0	0	15,120	77,590	69,296	65,037	70,873	151,473	277
Clannad: N-with	158,377	0	0	0	0	0	0	0	0	0	0	0	15,120	22,850	28,530	29,350	30,660	31,867	0 ^a^
Oxy with solid type	232,457	0	0	0	0	0	0	0	0	0	0	0	0	54,740	40,766	35,687	39,852	61,412	0
Oxy: Gaseupgi Dangbun	749,986	0	NA	NA	NA	NA	NA	NA	0	0	0	0	0	0	0	0	0	0	0
LG: 119 segyunjegeo	1,104,000	0	0	0	NA	NA	NA	NA	NA	NA	NA	0	0	0	0	0	0	0	0
Ato Safe	2353	0	0	0	0	0	0	0	0	0	0	0	0	0	0	0	361	1764	228
Eco-fresh: Hangkyunball	479	0	0	0	0	0	0	0	0	0	0	0	0	0	0	0	0	430	49
Water&People: Modern Life	56,000	0	0	0	0	0	0	0	0	0	0	0	0	0	0	0	0	56,000	0

^a^ Assigned “0” as the value when the number of annual recalls was larger than the number of annual sales. ^b^ Annual data were not available. PHMG, polyhexamethylene guanidine; CMIT/MIT, chloromethylisothiazolinone/methylisothiazolinone; PGH, oligo(2-(2ethoxy)-ethoxyethyl) guanidine chloride.

**Table 2:** Annual trends in the incidence of NHIS-recorded cases of J68.4, J84.9, J95.2, and S27.3 in South Korea during 2002–2017.

Diseases	Annual Incidence, Cases/100,000 Population															
	2002 ^a^	2003	2004	2005	2006	2007	2008	2009	2010	2011	2012	2013	2014	2015	2016	2017
J68.4 (chronic respiratory conditions due to chemicals, gases, fumes, and vapors)																
Overall	0.06	0.09	0.11	0.11	0.10	0.09	0.11	0.14	0.16	0.29	0.26	0.27	0.23	0.30	0.47	0.29
	(28) ^b^	(42)	(54)	(53)	(49)	(45)	(54)	(72)	(79)	(147)	(130)	(134)	(119)	(151)	(240)	(149)
Sex																
Male	0.07	0.11	0.12	0.12	0.13	0.09	0.16	0.16	0.20	0.34	0.29	0.33	0.28	0.31	0.67	0.30
Female	0.05	0.06	0.10	0.10	0.07	0.09	0.06	0.13	0.12	0.24	0.23	0.20	0.19	0.28	0.27	0.28
Age																
0–6 years	0.05	0.02	0	0	0.03	0.06	0.03	0.15	0.06	0.19	0.06	0.21	0.15	0.13	0.41	0.36
7–19 years	0	0.01	0.03	0.02	0.07	0.02	0.05	0.02	0.02	0.11	0.03	0.08	0.01	0.08	0.16	0.22
20–64 years	0.04	0.07	0.12	0.12	0.09	0.10	0.10	0.15	0.15	0.24	0.25	0.22	0.21	0.28	0.30	0.22
≥65 years	0.30	0.39	0.29	0.27	0.22	0.17	0.26	0.25	0.41	0.74	0.58	0.63	0.54	0.54	1.31	0.51
J84.9 (interstitial pulmonary disease, unspecified)																
Overall	10.56	11.47	12.95	14.49	16.89	18.00	18.45	19.78	20.55	23.40	22.77	23.24	25.49	29.05	34.73	34.13
	(5083) ^b^	(5540)	(6281)	(7056)	(8255)	(8845)	(9114)	(9824)	(10,248)	(11,728)	(11,465)	(11,748)	(12,942)	(14,800)	(17,751)	(17,483)
Sex																
Male	11.74	12.92	14.62	16.47	19.17	20.39	21.07	23.17	24.19	27.53	26.73	27.65	29.27	33.83	39.41	40.30
Female	9.38	10.01	11.28	12.50	14.59	15.60	15.81	16.39	16.89	19.27	18.81	18.82	21.72	24.27	30.06	27.97
Age																
0–6 years	2.32	2.41	3.21	4.43	6.55	5.52	6.65	10.11	7.12	12.09	9.82	3.25	4.17	8.38	7.23	3.00
7–19 years	0.64	0.57	0.78	0.99	1.07	1.06	1.33	1.52	1.04	2.03	1.30	0.99	1.75	2.13	3.20	2.04
20–64 years	8.12	8.17	9.04	9.72	10.58	11.53	11.63	11.64	12.82	14.09	12.38	12.96	14.55	15.54	19.52	17.91
≥65 years	54.42	60.82	65.95	72.10	83.93	84.60	82.64	87.69	86.48	94.67	96.01	95.14	97.27	108.57	121.32	120.74
J95.2 (acute pulmonary insufficiency following non-thoracic surgery)																
Overall	0.21	0.16	0.22	0.12	0.09	0.10	0.27	0.23	0.15	0.13	0.11	0.09	0.23	0.33	0.24	0.22
	(100) ^b^	(76)	(106)	(60)	(42)	(50)	(134)	(116)	(75)	(64)	(56)	(44)	(117)	(172)	(126)	(112)
Sex																
Male	0.16	0.13	0.25	0.13	0.09	0.11	0.23	0.21	0.16	0.10	0.09	0.08	0.23	0.39	0.26	0.23
Female	0.25	0.18	0.19	0.12	0.09	0.09	0.31	0.26	0.14	0.16	0.14	0.09	0.23	0.29	0.23	0.21
Age																
0–6 years	0	0	0.15	0.03	0	0	0.03	0.06	0	0.03	0.03	0	0.15	0.03	0	0
7–19 years	0.08	0.02	0.06	0.02	0	0	0.02	0.03	0.06	0	0.01	0	0.04	0.04	0	0
20–64 years	0.19	0.14	0.15	0.08	0.06	0.07	0.16	0.13	0.07	0.07	0.10	0.07	0.18	0.25	0.14	0.15
≥65 years	0.80	0.63	0.94	0.60	0.45	0.51	1.29	1.09	0.70	0.58	0.29	0.28	0.60	1.00	0.90	0.67
S27.3 (other injuries of lungs)																
Overall	0.74	1.22	1.60	1.73	1.60	0.98	0.64	0.64	0.63	0.13	0.07	0.05	0.02	0.03	0.02	0.01
	(358) ^b^	(585)	(771)	(834)	(771)	(470)	(309)	(310)	(302)	(63)	(33)	(22)	(12)	(14)	(8)	(4)
Sex																
Male	1.11	1.84	2.44	2.51	2.40	1.47	0.90	0.88	0.88	0.15	0.08	0.08	0.04	0.03	0.02	0.02
Female	0.37	0.58	0.73	0.91	0.75	0.44	0.35	0.37	0.33	0.10	0.05	0.01	0.01	0.02	0.01	0
Age																
0–6 years	0.39	0.72	0.55	0.87	0.31	0.49	0.09	0.37	0.12	0.03	0	0	0	0	0	0
7–19 years	0.34	0.51	0.68	0.71	0.41	0.49	0.32	0.30	0.24	0.02	0.01	0	0	0	0	0.01
20–64 years	0.81	1.28	1.72	1.76	1.74	0.92	0.68	0.68	0.61	0.13	0.07	0.04	0.02	0.03	0.02	<0.01
≥65 years	1.32	2.29	2.96	3.41	3.04	1.97	0.94	0.80	1.20	0.25	0.10	0.13	0.05	0.04	0.01	0.02

^a^ Cases in 2002 may include pre-existing cases. ^b^ Number of reported cases. Details of cases are described in Supplementary Table S1 online. NHIS, National Health Insurance Service

**Table 3:** Spearman’s rank correlation coefficient between NHIS-recorded HDLI cases (S27.3) and annual HD sales.

HD Sales	Lag Time (Years)					
	0	1	2	3	4	5
Total	0.907 ***	0.796 ***	0.649 **	0.419	0.143	−0.152
PHMG chemicals	0.952 ***	0.889 ***	0.756 ***	0.506 *	0.203	−0.105
CMIT/MIT chemicals	0.901 ***	0.781 ***	0.644 **	0.412	0.146	−0.143

* *p*-value < 0.05; ** *p*-value < 0.01; *** *p*-value < 0.001. NHIS, National Health Insurance Service; HD, humidifier disinfectant; HDLI, humidifier disinfectant-associated lung injury; PHMG, polyhexamethylene guanidine; CMIT/MIT, chloromethylisothiazolinone/methylisothiazolinone; PGH, oligo-(2-(2ethoxy)-ethoxyethyl) guanidine chloride.

**Table 4:** Participants’ characteristics of self-reported HDLI cases by survival status (*n* = 6059) ^a^.

Characteristics	Overall		HDLI-Survivors		HDLI-Deaths		*p*-Value ^b^
	N	(%)	N	(%)	N	(%)	
Total	6059	(100)	4754	(78.5)	1305	(21.5)	
Sex							<0.001
Male	3102	(51.2)	2444	(51.4)	658	(50.4)	
Female	2927	(48.3)	2310	(48.6)	617	(47.3)	
Unknown	30	(0.5)	0	(0.0)	30	(2.3)	
Age							<0.001
0–6 years	512	(8.5)	233	(4.9)	279	(21.4)	
7–19 years	1533	(25.3)	1503	(31.6)	30	(2.3)	
20–64 years	2950	(48.7)	2476	(52.1)	474	(36.3)	
≥65 years	1062	(17.5)	542	(11.4)	520	(39.9)	
Unknown	2	(0.0)	0	(0.0)	2	(0.1)	
Education level							<0.001
Elementary school	1990	(32.8)	1483	(31.2)	507	(38.9)	
Middle school	617	(10.2)	445	(9.4)	172	(13.1)	
High school	1336	(22.1)	1018	(21.4)	318	(24.4)	
College level or higher	1817	(30.0)	1591	(33.4)	226	(17.3)	
Unknown	299	(4.9)	217	(4.6)	82	(6.3)	
Humidifier disinfectant product name ^c^							<0.001
Oxy: New Gaseupgi Dangbun	4599	(75.9)	3631	(76.4)	968	(74.2)	
Lotte: WiseLect	126	(2.1)	101	(2.1)	25	(1.9)	
Homeplus: Chungjungje	93	(1.5)	77	(1.6)	16	(1.2)	
Homecare: Bejiteobeulhom	49	(0.8)	46	(1.0)	3	(0.2)	
Aekyung: Gaseupgi Mate	514	(8.5)	438	(9.2)	76	(5.8)	
E-Mart: Eplus	107	(1.8)	92	(1.9)	15	(1.2)	
GS: Hambakwussom	8	(0.1)	6	(0.2)	2	(0.2)	
SK: Yugong	15	(0.3)	13	(0.3)	2	(0.2)	
Butterfly Effect: Cefu	99	(1.6)	81	(1.7)	18	(1.4)	
Clannad: N-with	45	(0.7)	42	(0.9)	3	(0.2)	
Oxy: Gaseupgi Dangbun	51	(0.8)	44	(0.9)	7	(0.5)	
Others	35	(0.6)	30	(0.6)	5	(0.4)	
Unknown	318	(5.3)	153	(3.2)	165	(12.6)	

^a^ Self-reported case of HDLI. ^b^ *p*-value based on the Chi-square test. ^c^ Most frequently used product. HDLI, humidifier disinfectant-associated lung injury.

**Table 5:** Spearman’s rank correlation coefficient between self-reported HDLI cases and annual HD sales.

**(a) Self-Reported Cases of HDLI-Survivors**						
**HD Sales**	**Lag Time (Years)**					
	**0**	**1**	**2**	**3**	**4**	**5**
Total	0.711 ***	0.821 ***	0.871 ***	0.921 ***	0.901 ***	0.831 ***
PHMG chemicals	0.711 ***	0.811 ***	0.851 ***	0.881 ***	0.851 ***	0.781 ***
CMIT/MIT chemicals	0.621 ***	0.731 ***	0.791 ***	0.841 ***	0.821 ***	0.741 ***
** (b) Self-Reported Cases of HDLI-Deaths**						
**HD Sales**	**Lag Time (Years)**					
	**0**	**1**	**2**	**3**	**4**	**5**
Total	0.378	0.551 **	0.671 ***	0.761 ***	0.841 ***	0.891 ***
PHMG chemicals	0.421 *	0.581 **	0.671 ***	0.761 ***	0.841 ***	0.881 ***
CMIT/MIT chemicals	0.310	0.481 *	0.631 ***	0.721 ***	0.801 ***	0.771 ***

* *p*-value < 0.05; ** *p*-value < 0.01; *** *p*-value < 0.001. HD, humidifier disinfectant; HDLI, humidifier disinfectant-associated lung injury; PHMG, polyhexamethylene guanidine; CMIT/MIT, chloromethylisothiazolinone/methylisothiazolinone; PGH, oligo-(2-(2ethoxy)-ethoxyethyl) guanidine chloride.

## Data Availability

All the data and analysis material will be available online before the first online publication date.

## Supplementary Materials

The following are available online at https://www.mdpi.com/article/10.3390/ijerph18116136/s1: Table S1: Annual trends in the absolute number of NHIS-recorded cases of J68.4, J84.9, J95.2, and S27.3 in South Korea during 2002–2017, Figure S1: Flow diagram of the selection of the number of HD products, Figure S2: Annual sales of humidifier disinfectants and NHIS-recorded cases of J68.4, Figure S3: Annual sales of humidifier disinfectants and NHIS-recorded cases of J84.9, Figure S4: Annual sales of humidifier disinfectants and NHIS-recorded cases of J95.2.
