# Supplementary material for: Humidifier Disinfectant Consumption and Humidifier Disinfectant-Associated Lung Injury in South Korea: A Nationwide Population-Based Study
Source: Int J Environ Res Public Health. 2021 Jun 6;18(11):6136. doi: 10.3390/ijerph18116136 (PMC8201190; doi:10.3390/ijerph18116136)
Supplement: Supplementary file 1 [file ijerph-18-06136-s001.zip › Table S1.pdf]

Supplementary Table S1. Annual trends in the absolute number of NHIS-recorded cases of J68.4, J84.9, J95.2, and S27.3 in South Korea during 2002-2017

	Annual reported cases, N																
	Total	2002 [a]	2003	2004	2005	2006	2007	2008	2009	2010	2011	2012	2013	2014	2015	2016	2017
J68.4 (chronic respiratory conditions due to chemicals, gases, fumes, and vapors)																	
Overall	1,546	28	42	54	53	49	45	54	72	79	147	130	134	119	151	240	149
Sex																	
Male	927	17	27	30	29	31	22	39	39	49	86	73	84	72	79	172	78
Female	619	11	15	24	24	18	23	15	33	30	61	57	50	47	72	68	71
Age																	
0-6 years	62	2	1	0	0	1	2	1	5	2	6	2	7	5	4	13	11
7-19 years	72	0	1	3	2	6	2	4	2	2	9	2	6	1	6	11	15
20-64 years	881	13	22	37	37	30	31	33	49	48	81	84	73	70	96	101	76
≥65 years	531	13	18	14	14	12	10	16	16	27	51	42	48	43	45	115	47
J84.9 (interstitial pulmonary disease, unspecified)																	
Overall	168,163	5,083	5,540	6,281	7,056	8,255	8,845	9,114	9,824	10,248	11,728	11,465	11,748	12,942	14,800	17,751	17,483
Sex																	
Male	97,315	2,835	3,130	3,555	4,021	4,699	5,021	5,216	5,763	6,041	6,904	6,732	6,991	7,427	8,612	10,060	10,308
Female	70,848	2,248	2,410	2,726	3,035	3,556	3,824	3,898	4,061	4,207	4,824	4,733	4,757	5,515	6,188	7,691	7,175
Age																	
0-6 years	3,241	102	101	128	168	236	190	221	329	229	388	318	106	135	268	229	93
7-19 years	1,773	57	50	68	86	93	92	115	130	88	167	104	76	131	154	224	138
20-64 years	66,157	2,538	2,579	2,873	3,107	3,401	3,721	3,771	3,801	4,215	4,668	4,130	4,351	4,913	5,277	6,670	6,142
≥65 years	96,992	2,386	2,810	3,212	3,695	4,525	4,842	5,007	5,564	5,716	6,505	6,913	7,215	7,763	9,101	10,628	11,110
J95.2 (acute pulmonary insufficiency following non-thoracic surgery)																	
Overall	1,450	100	76	106	60	42	50	134	116	75	64	56	44	117	172	126	112
Sex																	
Male	709	39	32	60	31	21	28	57	51	41	24	22	20	59	99	66	59
Female	741	61	44	46	29	21	22	77	65	34	40	34	24	58	73	60	53
Age																	
0-6 years	18	0	0	6	1	0	0	1	2	0	1	1	0	5	1	0	0
7-19 years	33	7	2	5	2	0	0	2	3	5	0	1	0	3	3	0	0
20-64 years	657	58	45	49	26	18	21	53	42	24	23	33	23	61	84	47	50
≥65 years	742	35	29	46	31	24	29	78	69	46	40	21	21	48	84	79	62
S27.3 (other injuries of lung)																	
Overall	4,866	358	585	771	834	771	470	309	310	302	63	33	22	12	14	8	4
Sex																	
Male	3,637	269	445	594	613	589	362	223	219	219	38	20	20	9	8	5	4
Female	1,229	89	140	177	221	182	108	86	91	83	25	13	2	3	6	3	0
Age																	
0-6 years	150	17	30	22	33	11	17	3	12	4	1	0	0	0	0	0	0
7-19 years	353	30	45	59	62	36	43	28	26	20	2	1	0	0	0	0	1
20-64 years	3,372	253	404	546	564	560	297	221	221	199	43	25	12	8	11	7	1
≥65 years	991	58	106	144	175	164	113	57	51	79	17	7	10	4	3	1	2

[a] Cases in 2002 may include pre-existing cases; NHIS, National Health Insurance Service.
