# Supplementary figures and images for: Humidifier Disinfectant Consumption and Humidifier Disinfectant-Associated Lung Injury in South Korea: A Nationwide Population-Based Study
Source: Int J Environ Res Public Health. 2021 Jun 6;18(11):6136. doi: 10.3390/ijerph18116136 (PMC8201190; doi:10.3390/ijerph18116136)

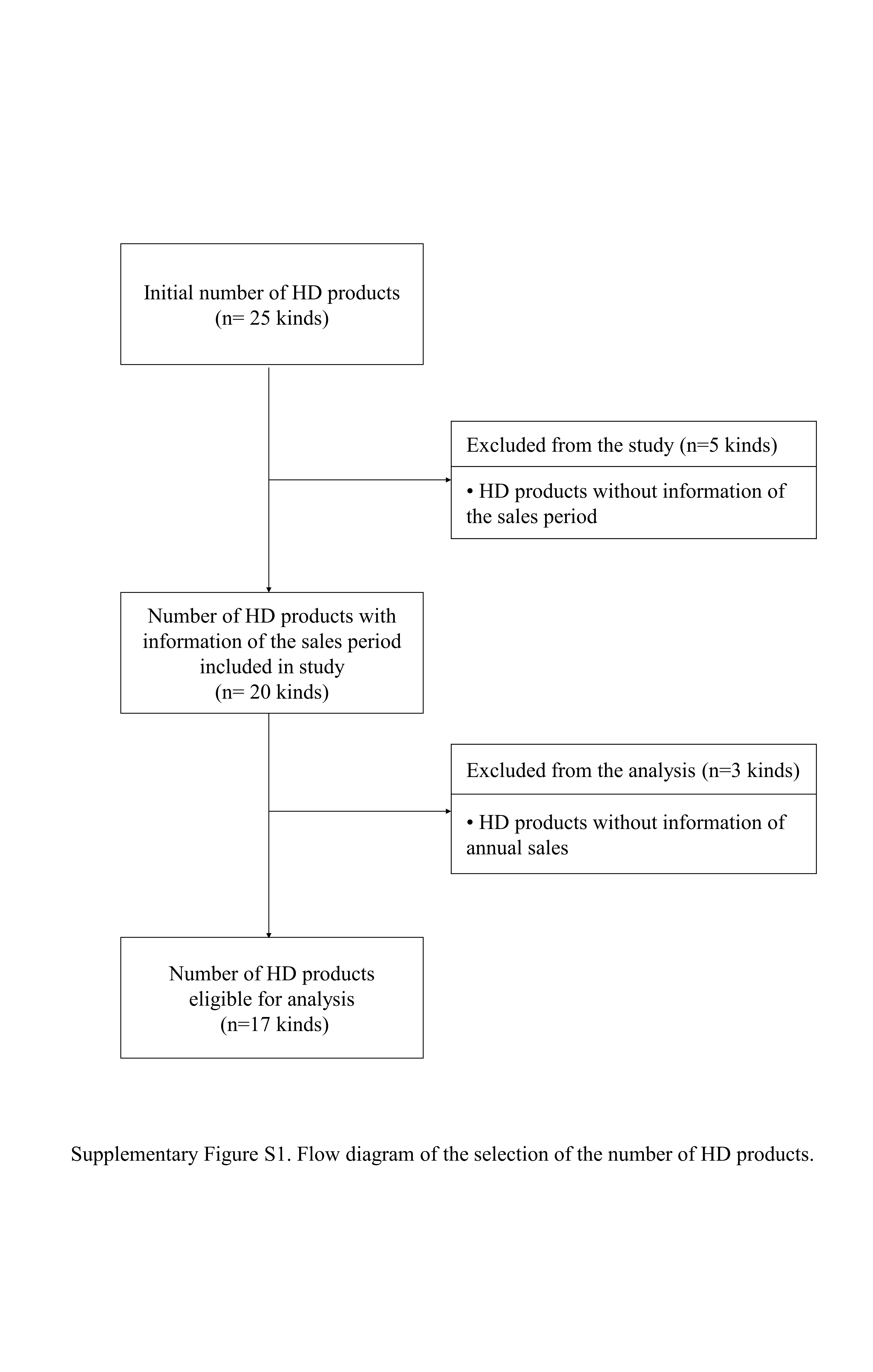

Supplement: Supplementary file 1 [file ijerph-18-06136-s001.zip › Figure S1.tif]

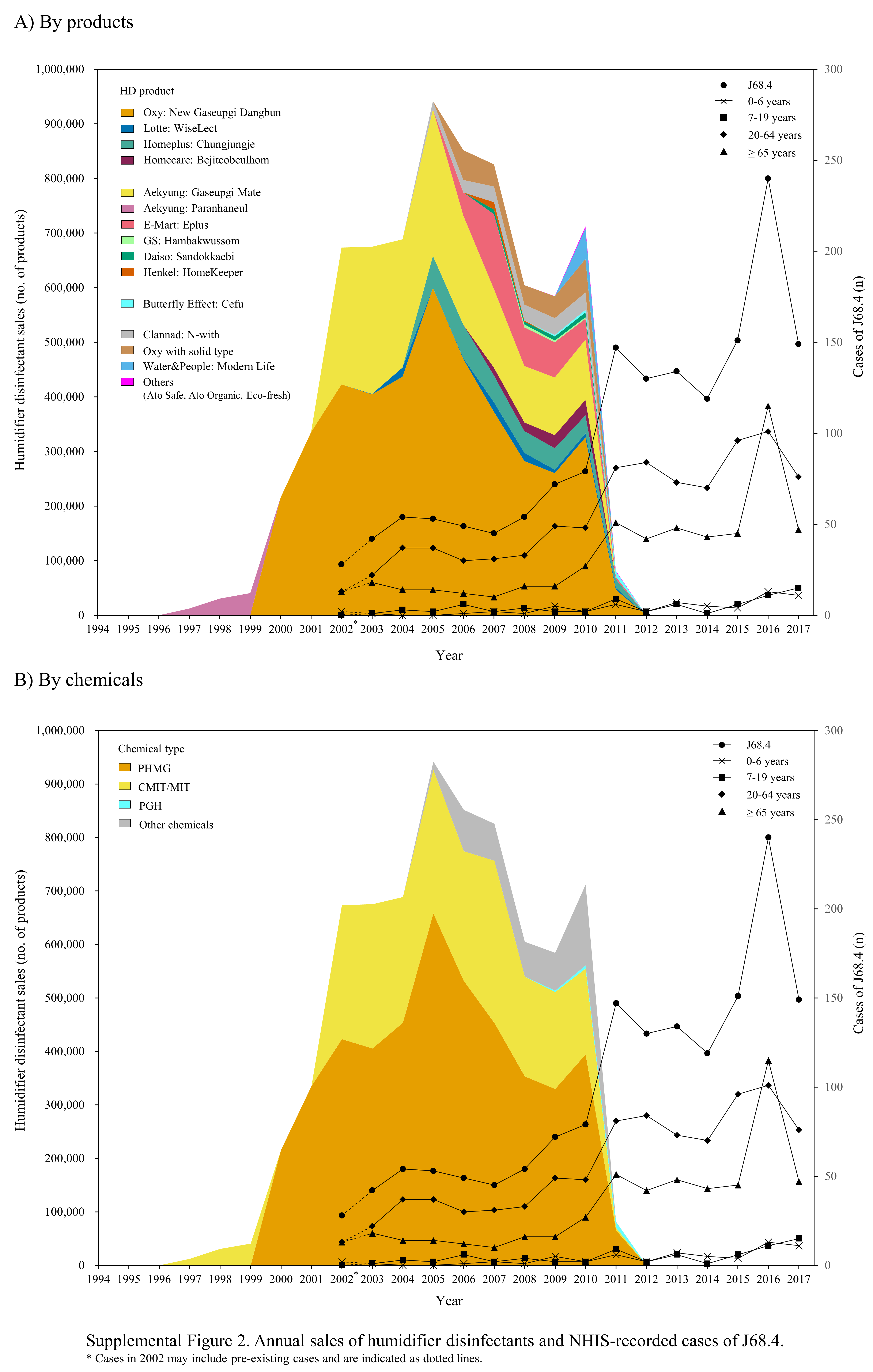

Supplement: Supplementary file 1 [file ijerph-18-06136-s001.zip › Figure S2.tif]

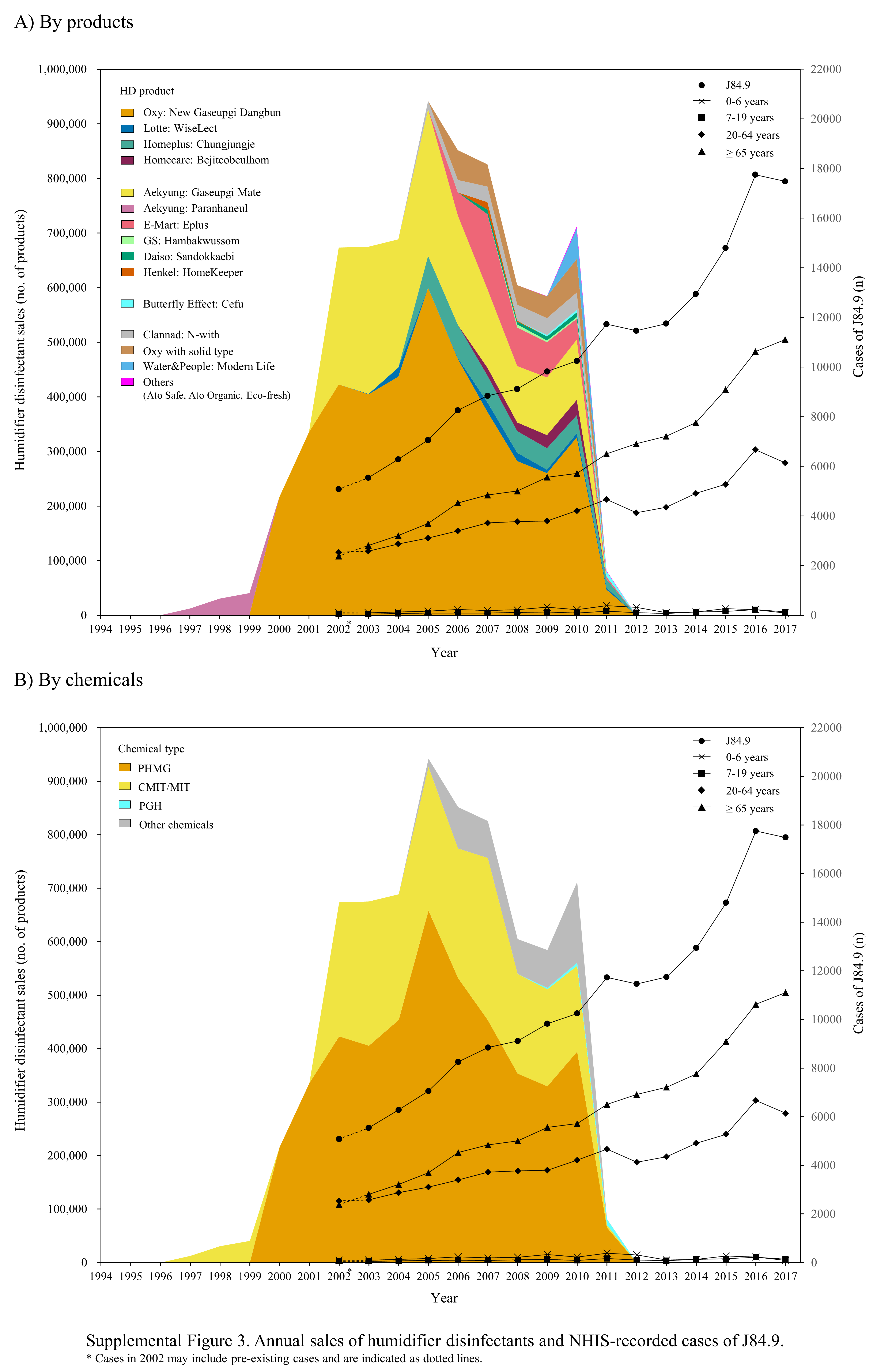

Supplement: Supplementary file 1 [file ijerph-18-06136-s001.zip › Figure S3.tif]

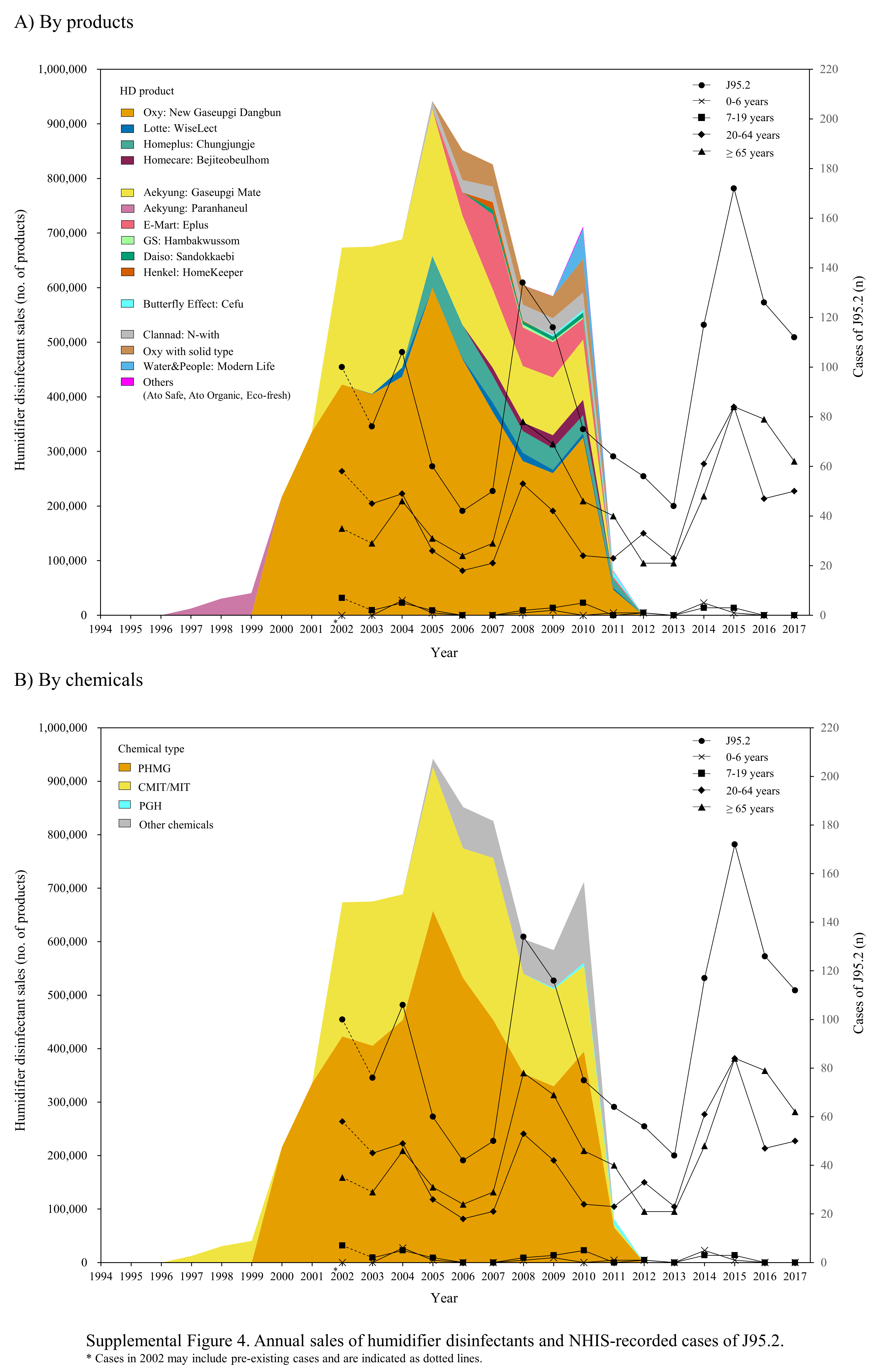

Supplement: Supplementary file 1 [file ijerph-18-06136-s001.zip › Figure S4.tif]
